# Integrating mission-aligned value with cost to assess the economic impact of AI in healthcare

**DOI:** 10.1038/s41746-026-02892-z

**Published:** 2026-06-11

**Authors:** Arwen BL Declan, R. Andrew Taylor

**Affiliations:** 1 Department of Emergency Medicine, Prisma Health—Upstate, Greenville, SC USA; 2 Clemson University, Clemson, SC USA; 3 University of South Carolina School of Medicine Greenville, Greenville, SC USA; 4 Department of Emergency Medicine, University of Virginia, Charlottesville, VA USA

**Keywords:** Business and industry, Health care, Mathematics and computing

## Abstract

Healthcare organizations must integrate mission-driven values with financial metrics to govern AI systems. We propose the Total Mission Value (TMV) framework, drawing on insights from Balanced Scorecard, Quintuple Aim, and organizational mission priorities. TMV integrates five ethically grounded domains that define TMV in healthcare: Patient Care, Staff Experience, Operations, Economic, and Education and Research. TMV supports aligning AI implementations with healthcare’s core purpose while maintaining economic sustainability.

## Introduction

As Artificial Intelligence (AI) technologies advance in healthcare, organizations must align AI governance with healthcare’s core mission, which is rooted in ethical obligations of beneficence, non-maleficence, justice, and respect for autonomy^1,2^. These obligations extend beyond individual care to institutional decisions, ensuring AI technologies benefit patients, communities, and the workforce, not just financial gains. AI technologies, from focused-use tools to autonomous multiagent systems, appear poised to transform healthcare^3–7^. Yet the scope and velocity of AI adoption amplify the ethical stakes of governance decisions. AI tools could enhance care speed, diagnostic accuracy, and costs efficiency while supporting population health, scientific inquiry, and operational management. However, they also introduce risks of bias, opacity, workforce displacement, and erosion of the patient-clinician relationship that are invisible to cost-focused analyses^8,9^. Governing AI’s economic impact without integrating mission-driven values thus risks both strategic misalignment and ethical failure.

AI governance depends on strategic business decisions that require understanding AI’s economic and mission-congruent value. Integrating economic impact with mission-congruent values supports organizational financial and mission alignment^10,11^. Since healthcare is uniquely motivated by its intrinsic mission, strategic business decisions in healthcare must reflect mission-defined values in addition to economic impact. However, AI economic analyses in healthcare often focus on costs, not mission-drivenvalues^1,12–15^. Focusing primarily on costs decouples strategic business decisions about technology adoption from an organization’s core mission, creating strategic misalignment, excluding stakeholder values, and diminishing performance outcomes^11^. Aligning mission-driven value with financial metrics supports mission-congruent strategic decisions that are normatively essential and overall empirically beneficial for governance across the AI lifecycle.

Given the importance of aligning mission-driven values with financial outcomes for strategic decision-making, we propose an integrative approach to value-driven governance for AI in healthcare. We define a conceptual framework, Total Mission Value (TMV), that integrates mission-driven value with cost-focused analyses for strategic AI governance decisions. TMV is not a quantitative composite index or a replacement for existing health economic evaluations; it integrates mission-aligned value with financial metrics for strategic organizational decisions about AI governance. TMV is motivated by the normative perspective that mission-value alignment is essential and, as evidenced by the broader business literature, empirically associated with stronger organizational performance. To define TMV’s value domains, we draw on the Balanced Scorecard (BSC) approach to mission-value integration from the business literature, analyze the Institute for Healthcare Improvement’s (IHI) Quintuple Aim for broad healthcare goals, and examine Mission, Vision, and Values (MVV) statements for organizational strategic priorities. This analysis reveals five ethically grounded value domains that define TMV in healthcare: Patient Care, Staff Experience, Operations, Economic, and Education and Research. These domains build the foundation for future work to define quantifiable outcome metrics for AI technologies, develop TMV scorecards, and implement TMV for strategic governance across the AI lifecycle.

## Mission-value alignment across the broader business context

The case for integrating healthcare’s mission-driven value with economic outcomes for AI governance is fundamentally normative, though well supported by empirical observations. Organizational mission, vision, and values are critical for high-impact AI governance decisions. As AI technologies emerge, existing normative and empirical strategic insights can inform healthcare’s approach to AI governance. We summarize insights from the business literature on strategic IT decisions, highlighting mission-value alignment and pragmatic approaches for defining value for strategic business decisions. These insights provide a foundation for defining TMV for AI governance in healthcare.

### Mission-value alignment is essential for strategic business decisions about IT

Organizational alignment entails agreement on a shared purpose, typically encapsulated in an organization’s MVV statement. Effective alignment requires aligning decisions with organizational MVV, a widely acknowledged necessity^10,11,16,17^. Empirical studies of IT adoption across industries reinforce this perspective. IT influences organizational structure and capabilities, creating opportunities for value creation^18–21^. Prioritizing customer satisfaction enhances financial performance^22^. Incorporating non-financial metrics into organizational strategic business outcomes is associated with higher performance compared to focusing solely on financial metrics^23^. Pursuing socially- and environmentally-driven organizational practices through B-corp certification is associated with higher financial performance than that observed in traditional companies^24^. Although some studies describe a paradox, where strategic mission-value alignment is negatively correlated with organizational performance, further analyses suggest that these are due to flawed methods or conceptual frameworks^10,25^. Given the substantial empirical evidence connecting strategic mission-value alignment with enhanced organizational performance across industries, healthcare organizations should integrate mission-driven values into their strategic AI governance.

### The Balanced Scorecard approach models mission-value alignment across industries

To integrate mission-driven value with financial metrics for strategic AI decisions, we draw on the Balanced Scorecard approach. The BSC combines financial outcomes with organizational values by defining goals and outputs aligned with an organization’s mission^26^.Compared to other performance-focused business approaches and decision tools like Multi-Criteria Decision Analysis (MCDA) and health economic analyses, the BSC uniquely integrates mission-driven values, strategic priorities, and metrics within a framework applicable across an organization^1,14,27–30^. The BSC is widely used across industries, including IT and healthcare, and is linked to significant benefits across financial and mission-congruent performance^19,31–36^. It offers an ideal structure for TMV’s domains of value for AI governance in healthcare.

**Fig. 1:**
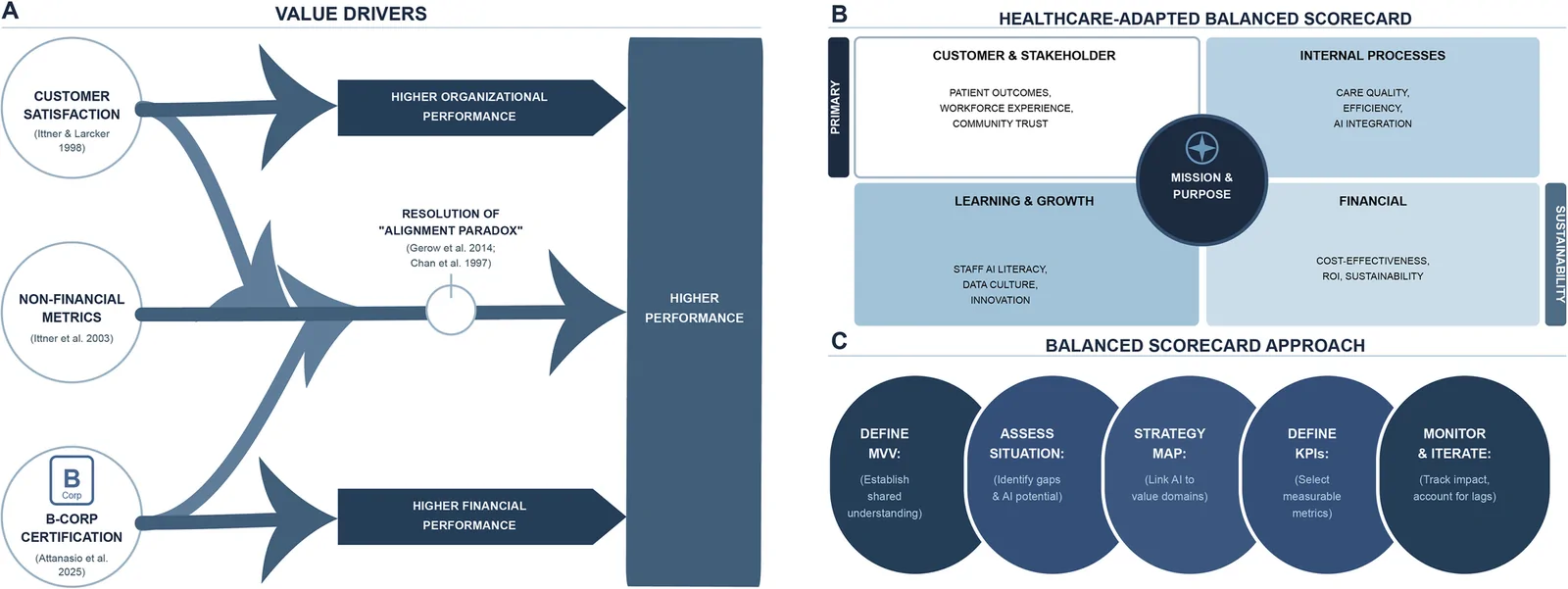
A strategic framework for mission-value alignment in healthcare AI adoption. **A** Empirical evidence supports integrating non-financial value with financial outcomes. The “alignment paradox,” in which mission-value alignment appears negatively correlated with performance, is likely due to methodological flaws rather than a true inverse relationship. **B** The Balanced Scorecard (BSC) adapted for healthcare, centering on mission. Customer and stakeholder perspectives (patients, workforce, and community) are prioritized; financial metrics ensure sustainability. Internal processes and learning and growth complete the framework. **C** The BSC implementation lifecycle for AI adoption decisions involves iterative steps: defining organizational mission, vision, and values (MVV); situation assessment; strategic mapping; Key Performance Indicator (KPI) definition; and ongoing monitoring and feedback.

The BSC encapsulates value by mapping priority categories to domains and Key Performance Indicators (KPIs; Fig. 1A). The original BSC prioritized financial, customer, business, and learning/growth categories^26^. In healthcare, these domains shift: learning and growth drives processes and impacts care quality; customer perspectives outweigh financial metrics; and the customer domain expands to include patients, the workforce, payers, and the community (Fig. 1B)^31,35,37^.

A BSC can be developed across an organization, within a unit, or collaboratively across units. Developing and using a BSC begins with clearly defining organizational MVV (Fig. 1C)^35,36^. Leaders must establish a shared understanding of MVV as the BSC’s foundation. They assess the situation, identify desired changes, and create a strategic map linking value domains with goals, processes, and performance metrics. This map illustrates key cause-and-effect relationships for selecting relevant outcome metrics, such as KPIs^38,39^. Quantifying IT’s value is difficult^18^, but the BSC’s mission-driven scorecard approach helps define KPIs that reflect IT’s strategic, mission-congruent value. As internal process drivers, AI technologies also are difficult to value due to indirect links between implementation and performance metrics, focus on financial outcomes, measurement challenges, environments, and time lags between adoption and outcomes^18^. These challenges can be addressed by mapping values, strategic priorities, and outcomes in a disciplined BSC approach. Thus, defining mission-driven value through an integrated scorecard framework clarifies value analyses for AI governance decisions.

Developing a BSC framework requires disciplined definition of values, goals, and performance metrics. A high-impact BSC framework must link outcome metrics with processes, align with goals and performance drivers, be independent of resources, and be strategic rather than tactical^35,36^. Failure to meet these criteria suggests inadequate organizational maturity for IT integration. Consequently, the scorecard approach can provide a critical checkpoint for integrating new, governance-intensive IT like AI: organizations unable to define a scorecard for AI may need to postpone adoption. After developing a BSC, organizations must ensure effective communication, dissemination, and management processes that integrate business planning and serial feedback for learning^35^. A disciplined BSC approach can yield insights and beneficial impact for organizations aligning strategic decisions with mission-driven values.

## Defining TMV’s domains of value for AI in healthcare

To define TMV domains for AI governance in healthcare, it is essential to connect BSC domains with healthcare’s mission-driven values, creating a foundational value framework within which organizations can tailor strategic priorities and measurable outcomes for AI governance. Understanding healthcare’s mission-driven values, articulated in the IHI’s Quintuple Aim and in individual MVV statements, is essential. The broad scope of healthcare’s mission is articulated in the IHI’s Quintuple Aim, while the mission of an individual healthcare organization is typically encapsulated in a MVV summary statement. To build the foundation for defining TMV, we analyze value categories from the Quintuple Aim and organizational priorities from MVV summaries. By merging these organizational perspectives, we surface and integrate key domains of value to develop a conceptual approach to defining TMV for AI governance as mission-driven value in healthcare.

### The Quintuple Aim: Domains of healthcare’s mission

**Fig. 2:**
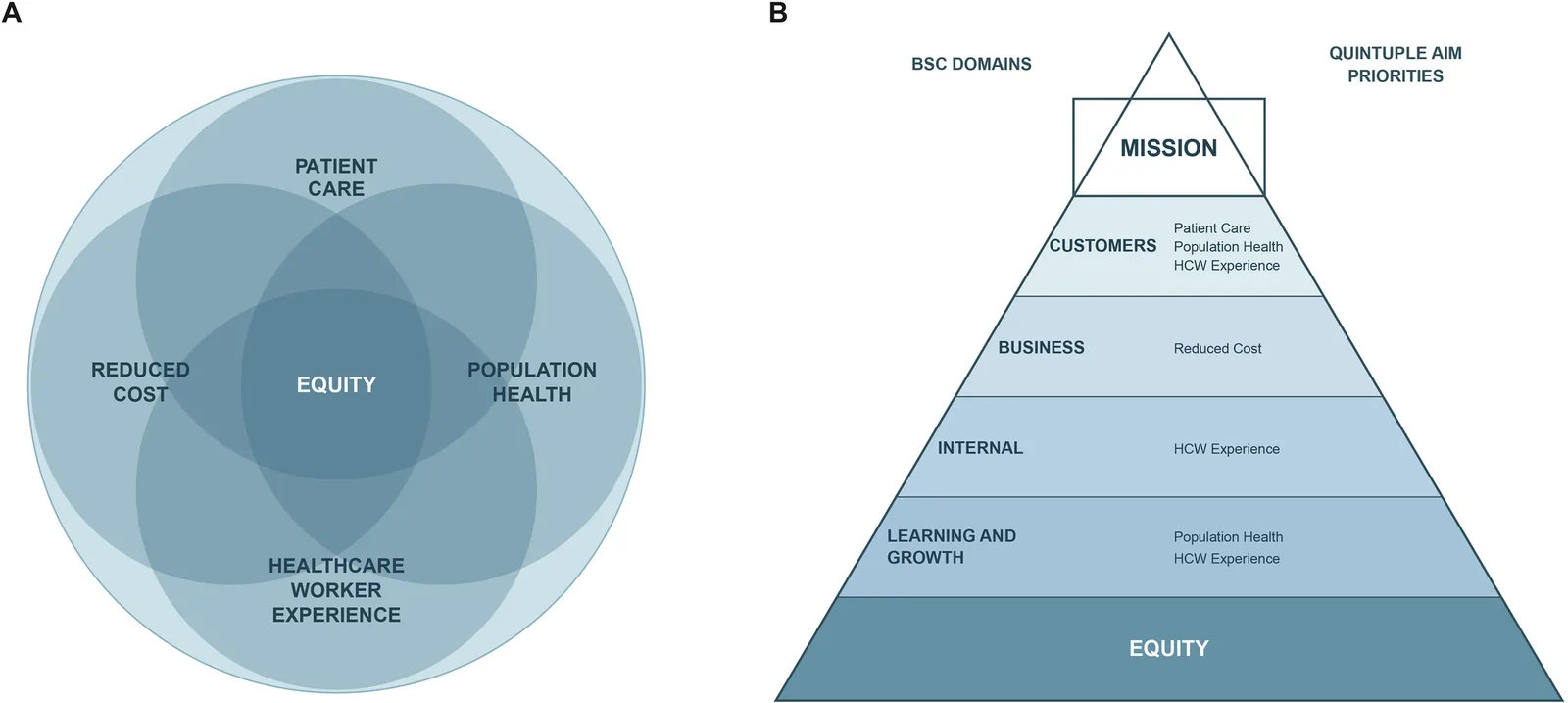
Integration of healthcare mission-driven values and balanced scorecard domains. **A** The core components of the Quintuple Aim—patient care, population health, reduced cost, and healthcare worker (HCW) experience—are represented as intersecting priorities. Health equity serves as the essential, unifying foundation that connects and informs all other domains. Adapted from Olayiwola & Rastetter (2021). **B** A conceptual framework mapping traditional Balanced Scorecard (BSC) domains (Customers, Business, Internal, Learning and Growth) to specific Quintuple Aim priorities. This alignment demonstrates how broad, mission-driven values are translated into actionable organizational goals to define Total Mission Value (TMV). Both models position equity as the underlying base supporting the overarching organizational mission. Adapted from Wu & Kuo (2012) and Voelker et al. (2001). HCW = healthcare worker.

Healthcare’s core goals, as summarized in the IHI’s Quintuple Aim, include patient experience, population health, reducing healthcare costs, healthcare worker (HCW) experience, and health equity (Fig. 2A)^40,41^. These goals represent broad conceptual categories that encompass surrogate value metrics. Patient experience includes patient satisfaction and engagement, while population health encompasses disease-specific outcomes. Efficiency and safety contribute to reducing healthcare costs, whereas communication, workload, and time spent with patients influence HCW experience. Equity, the fifth and newest aim, addresses ethical challenges including disparities and social determinants of health; equity is also an essential component of the other four aims. The Quintuple Aim domains do not strictly correspond to BSC categories (Fig. 2B), yet they provide essential insights into healthcare’s core mission-driven values. To understand how individual organizations operationalize these values, we examine organizational MVV statements for strategic priorities.

### Mission-driven values revealed by MVV

The common themes in organizational MVV statements reveal fundamental values across healthcare (Table 1)^42^. The strategic priorities summarized in MVV statements center on patient care as the defining mission and inspirational vision of healthcare^43^. Grouping these strategic priorities thematically by the BSC’s business categories and the Quintuple Aim’s goals, we identify five value domains: Patient Care^43–46^, Staff Experience^42,43^, Operations^42,43^, Economic^45,46^, and Education and Research^42,43,45^. MVV analyses also surface ethical considerations, including equity. Recognizing ethical conviction as the foundational inspiration for medical care, we emphasize its contribution across every value domain. We define TMV by these five ethically grounded domains.

**Table 1:** Values and strategic priorities revealed by Mission, Vision, and Values (MVV) statements of healthcare organizations

Value domain	Strategic priorities	Ethical foundations
Patient Care	Individualized respect for persons across personalization, quality of life, diversity, and holistic care including spiritual and emotional support. Patient-centered care: compassion, trust, participation in care. Care quality. Population health, public health, community and societal impact.	Integrity, honesty, confidentiality, respect, trust, compassion. Social responsibility (public health, societal impact). Equity: access, social determinants of health, diversity and inclusion.
Staff Experience	Workforce development and success. Staff feel valued. Teamwork and respectful collaboration across disciplines. Safe, supportive workplace with respect for diverse individuals and viewpoints.	Equitable treatment and inclusion across the workforce. Organizational cultures of respect that support well-being, development, and retention.
Operations	Resource management across financial and human capital. Quality indicators (clinical, performance). Risk management.	Responsible stewardship of resources and dedication to quality outcomes. Harm prevention (*primum non nocere*), including equitable risk mitigation. ***Note:*** *Risk management is underrepresented in MVV statements despite its significance*.
Economic	Capital management. Cost to patients. Organizational financial outcomes and resource allocation.	Cost to patients impacts access, compliance, and outcomes; patient cost may be under-evaluated in MVV statements. Tension between financial gain and mission-driven values may be reconciled through integrated frameworks.
Education & Research	Knowledge acquisition among staff and patients. Scholarly inquiry, discovery, creativity, and innovation. Ongoing education and evidence-based practice. Contributes to patient care, care quality, and internal processes.	Education supports equitable care through informed patients and competent staff. Research advances evidence-based practice and drives innovation across all value domains.

Strategic priorities identified from MVV analyses are grouped into five value domains that integrate Balanced Scorecard (BSC) business categories with Quintuple Aim goals. Although value domains do not strictly correspond with BSC (Customer, Business, Internal, Learning & Growth) or Quintuple Aim (patient experience, population health, reducing healthcare costs, provider experience, health equity) priorities, MVV value domains encompass both. Ethical foundations, including equity, suffuse each domain rather than constituting a separate category. In this framework, AI and other information technologies are represented within the Operations domain. Adapted from Williams et al.^43^, Qin et al.^42^, Bouvette et al.^45^, Cronin and Bolon^46^, and Iyiola et al. (2025).

#### Patient care

Consistent with healthcare’s fundamental aim to enhance health and treat disease, the central focus of MVV statements is commitment to patient care^43–46^. Quality of care also features prominently^43,46^, where quality is expressed as “meeting the physical needs of the patient” through “appropriate, prompt, and efficient care for the patients with respect to the medical treatment of their illness.”^43^ Commitment to patient care also includes respect for individuals, patient participation, personalization, patient-centered care, quality of life, and holistic care including spiritual and emotional support. These descriptors represent a broad range of ethical and equitable values that reflect healthcare’s fundamental ethical foundations^42,43,45,46^. Notably, patient-centeredness, an important contributor to high-quality care, is often emphasized^42^.

#### Staff experience

Staff experience in MVV statements reflects organizational cultures of respect^42,43^. Consistent with MVV statements that include *all* healthcare organizational staff rather than the Quintuple Aim’s more limited focus on healthcare workers, our value domain encompasses the experience of all of a healthcare organization’s workers. This approach is intentionally inclusive across the entire healthcare organization, recognizing the breadth of skills and service necessary for patient care. Cultures of respect are revealed by team collaboration, and are linked with respect and support for diverse individuals and viewpoints. Thus, equity toward staff is embedded in this domain. However, staff experience involves feeling valued and satisfied with development, compensation, and support; staff well-being is linked with professional success, education, and research and overlaps with education and research^47^.

#### Operations

Operations parallel the BSC’s internal process domain and include financial and human capital management^42,43^. Internal processes are implicit in the management of quality indicators across clinical and performance domains; thus, AI governance is incorporated into the operations domain. Notably, risk management, crucial for care quality and operations, is not well represented in MVV statements^43^. This absence is notable; risk management prevent and contributes significantly to care quality. Further, risk management is essential for responsible stewardship of ethical and equitable care^48^.

#### Economic

The Economic domain includes patient costs, organizational financial outcomes, and capital management^45,46^. Cost is a known barrier to care, a social determinant of health, and a negative predictor for health outcomes, yet patient cost is underrepresented in MVV statements^44^. Payers as stakeholders are generally included in this domain, though their interests may also be represented through quality and operational metrics. Hospital resource management and resource allocation are represented across MVV statements; financial outcomes for Health IT implementations have been well evaluated through economic impact analyses^1,12–15^, but hospital financial gain is only sporadically represented in MVV statements^43^. There may be inherent tension between hospital financial gain and mission-inspired values^43^, so healthcare organizations must address such conflicts. A methodical approach to integrating financial outcomes with mission-driven values helps identify conflicts and expands understanding of trade-offs reflecting stakeholder perspectives, organizational financial outcomes, and mission-driven strategic priorities.

#### Education and Research

Education and Research categories are well represented across healthcare MVV statements^42,43,45^. We modify the BSC’s “Learning and Growth” category to “Education and Research” to reflect healthcare’s reliance on education for clinical competence and on research for evidence-based practice^37^. Healthcare organizations have unique opportunities to educate patients and staff; education contributes to patient care, care quality, internal processes, and equity. Similarly, research and scholarly inquiry foster discovery, creativity, and innovation to enhance patient care.

**Fig. 3:**
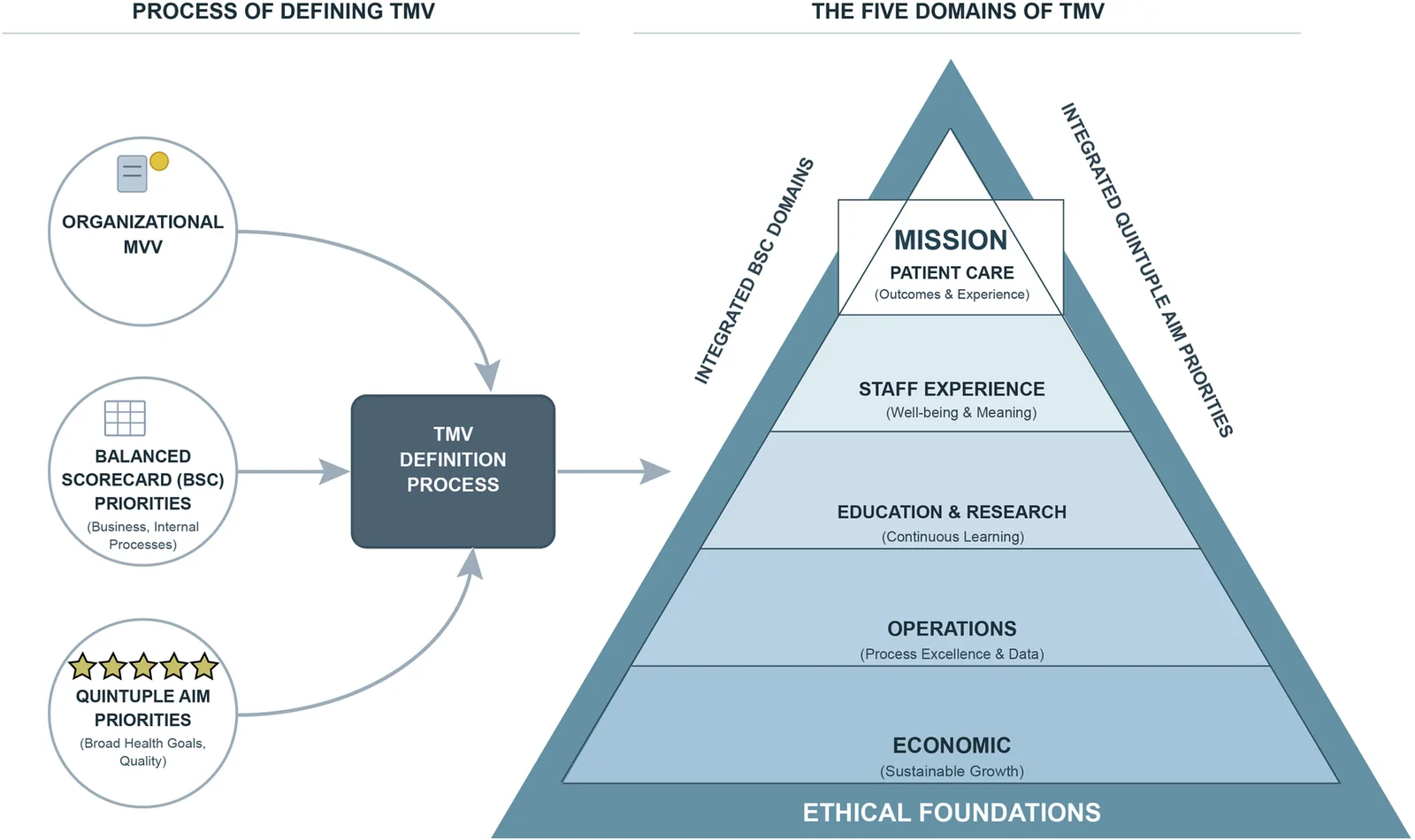
Total mission value (TMV). Domains of value for healthcare that integrate mission-aligned values with economic outcomes. Five ethically grounded value domains—Patient Care, Staff Experience, Operations, Economic, and Education and Research—define TMV by integrating business priorities from the Balanced Scorecard (BSC), broad healthcare goals from the Quintuple Aim, and organizational strategic priorities revealed by Mission, Vision, and Values (MVV) statements. Ethical foundations, including equity, suffuse each domain rather than constituting a separate category. Patient care occupies the central apex, consistent with its defining role in healthcare MVV statements, and extends to encompass population health and community impact. AI and other information technologies are represented within the Operations domain. The framework establishes a conceptual foundation for future work to define quantifiable outcome metrics and implement TMV scorecards for strategic AI adoption decisions. The right panel is adapted from BSC visualizations (e.g., Wu & Kuo^37^ Voelker et al.^35^).

Using these five domains (Patient care, Staff experience, Operations, Economic, and Education and Research), we integrate business priorities from the BSC, broad healthcare goals from the Quintuple Aim, and organizational values surfaced from MVV statements into the TMV scorecard on these domains of value (Fig. 3). Acknowledging healthcare’s ethical foundations, we integrate ethical foundations, including equity, throughout TMV^2,49^. Two adaptations from the BSC and Quintuple Aim categories merit further emphasis. First, recognizing patient care as healthcare’s central, defining mission across MVV statements, we separate the BSC’s stakeholder domain into Patient Care and Staff Experience. Second, we expand the Patient Care domain to encompass population health, public health, and community and societal impact within healthcare’s primary patient-centered mission. Organizations may categorize community and societal impact under other domains, but we consider them extensions of healthcare’s purpose. Together, these five ethically grounded domains establish the TMV’s conceptual foundation for mission-aligned AI governance in healthcare.

## TMV perspectives inform responses to challenges in AI deployment

The mission-aligned domains that comprise TMV provide insight into known challenges across the AI lifecycle. AI deployment faces challenges like model generalizability and bias risk, performance degradation, and regulatory uncertainty. Each of these challenges correlates with at least one core TMV domain; exploring these from the perspective of TMV reveals insights grounded in mission-driven value.

Model generalizability is a well-known challenge^50,51^. AI models trained in one population often do not generalize to another population. This lack of generalizability can introduce errors, including biases amplified from training data or resulting from inadequate population representation in training data^52,53^. Within the TMV framework, errors resulting from generalizability are operational priorities, since such errors risk care quality and expose patients to risk of harm: generalizability errors strategically map to erroneous output, leading to clinical errors. KPIs include disease-specific outcome metrics and treatment decisions.

Errors like bias also fall under the TMV Patient Care domain. Shifting from operational to patient-focused metrics broadens the strategic perspective on non-generalizability and bias. Equitable algorithmic output is essential for ensuring respect for persons, which supports patient participation, empowerment, trust of healthcare providers, and better health outcomes. Relevant KPIs include metrics from satisfaction with care (e.g., survey-based), personalized population health (e.g., wearable-tracked or self-reported activity logs), and patient engagement across diverse groups (e.g., portal access)^54^. Mapping patient-centered priorities to equitable algorithmic output reveals opportunities across the AI lifecycle for bias mitigation, equity-driven bias prevention and mitigation strategies, and impact tracking for initiatives^52^. Thus, TMV provides a conceptual framework for mitigating bias and ensuring equitable AI output.

AI performance degradation is a serious challenge within the TMV’s Operational domain. It occurs when an AI system’s performance worsens over time, largely due to model drift from changes in the input data over time; it may result from concept drift due to changes in contextual expectations^51,52^. Since drifting data and contextual expectations affect downstream quality metrics, key KPIs include clinical quality metrics and data stability. Monitoring drift across the AI lifecycle helps healthcare organizations identify key opportunities to retrain, replace, or sunset AI models for effective mission-driven AI governance^52^.

In the rapidly changing landscape of AI regulation^55^, regulatory uncertainty produces major Economic challenges for healthcare AI governance. External regulations affect reimbursement and financial risk, influencing AI governance through impact internal policies, internal processes, financial outcomes, and legal liabilities^56–58^. Regulatory uncertainty can cause financial loss and regulatory censure. Organizational KPIs may therefore reflect anticipated regulations and legal liability risks. To minimize risks induced by regulatory uncertainty, future-oriented AI governance can target quality, safety, and performance KPIs for high-risk AI tools^58^.

AI deployment challenges and associated costs span the AI lifecycle. Managing lifecycle costs, represented in TMV’s Economic domain, is crucial. Although calculating AI lifecycle costs in healthcare is difficult due to regulatory requirements such as auditing and data encryption^59^, cost-aware governance across the AI lifecycle is essential, especially given healthcare’s tight profit margins. Relevant KPIs across the AI lifecycle include costs of deployment, integration, monitoring, maintenance, and retraining, either individually or combined as an aggregate metric^59^. Mission-driven AI lifecycle cost management provides essential insights for strategic business decisions.

## Applying TMV: Case study of ambient dictation

We demonstrate TMV’s pragmatic application using ambient dictation as a case study. Within each domain of TMV, we describe key strategic priorities and relevant ethical considerations that define a hypothetical TMV scorecard (Table 2). We derive KPIs from recent literature on novel implementations of ambient dictation in the clinical environment.

**Table 2:** TMV Scorecard for an ambient dictation system

Value domain	Strategic priority	Related ethical topics	Potential KPIs
Patient Care	Restoring the patient-physician connection and enhancing clinical documentation quality.	• Patient privacy and data security regarding third-party audio processing • Informed consent	• Patient experience surveys: attention scores, e.g., “Doctor always listens carefully”
Staff Experience	Mitigating physician burnout, cognitive load, and administrative documentation burden.	• Staff feel valued and supported • Professional accountability and clinical judgment for the final medical record.	• Burnout prevalence (Mini Z) • Workload, e.g., NASA Task Load Index (NASA-TLX) • After-hours charting (“pajama time”)
Operations	Improving clinical workflow efficiency	• Clinician time management • Equity in AI performance for diverse patient populations (e.g., accents, dialects, or interpreters).	• Total time spent in the EHR per appointment or per note • Number of patient encounters per day or week • Same-day note completion rates.
Economic	Maximizing return on investment (ROI) through improved coding accuracy, billing integrity, and workforce retention.	• Accurately capturing care intensity versus the potential for AI to drive “upcoding”; • Medico-legal liabilities associated with discoverable raw audio recordings	• Work Relative Value Units (wRVUs) generated • Claim denial rates • Incremental revenue generated per provider • Avoided physician turnover costs
Education & Research	Optimizing workflow support through innovation; defining ambient dictation’s impact on clinician efficacy, care processes, and outcomes.	• Enhancing evidence-based practice and ethical care • Driving innovation to support workflows	• Learner metrics: core skill evaluation scores • Workflow evaluation, e.g., NASA-TLX

TMV domains guide mission-driven consideration ambient dictation toward quantifiable Key Performance Indicators (KPIs). Patient Care focuses on the patient-physician connection while respecting privacy and consent; patient-physician relational impact is measured through patient experience surveys. Staff Experience aims to reduce burnout and cognitive load, through established measurements like the Mini Z burnout survey and the NASA-TLX instrument. Operations seeks workflow efficiency and equity in AI performance, tracked by EHR time and encounter rates. In the Economic domain, ROI optimization through billing integrity and workforce retention is monitored by work Relative Value Units (wRVUs) and turnover costs. Education & Research emphasizes innovation and ethical practice, evaluated through learner metrics and workflow assessments.

Within the Patient Care domain, ambient dictation decreases charting time, allowing physicians to focus on patients^60,61^. This should restore patient-provider connections and enhance clinical documentation quality. Ethical concerns involve patient privacy, data security, and informed consent. Success can be measured by patient experience surveys that incorporate physician attention scores (e.g., increasing from 3.5 to 4.1 on a 5-point scale for the “Doctor always listens carefully” question) and patient engagement metrics^54,62^. Workflow changes should benefit Staff Experience by reducing physician burnout, cognitive load, and documentation burden^60,63^. Key ethical topics include ensuring staff feel valued and supported while maintaining professional accountability for the medical record^64,65^. Performance indicators include self-reported burnout prevalence (e.g., dropping from 51.9% to 38.8% using the Mini Z), workload measurement (e.g., NASA Task Load Index) and measured reductions in after-hours charting, or “pajama time”^61,63,64^.

Integrating patient- and staff-centered metrics addresses concerns that organizations will exclusively target AI-driven clinical productivity^65^. Combining these metrics with an Operational focus on clinical workflow efficiency helps align organizational, employee, and patient priorities. In addition to ethical time management, Operational goals must also target equitable ambient dictation implementation that serves diverse populations, including those with accents, dialects, or interpretation needs^61^. Relevant KPIs measure total Electronic Health Record (EHR) time per appointment, patient encounters per unit time, and same-day note completion rates^66,67^. Economic considerations aim to maximize Return on Investment (ROI) through improved coding accuracy, billing integrity, and workforce retention^62,66^. The primary ethical challenge is ensuring accurate documentation, balancing charge capture against AI-driven “upcoding.” Legal liabilities from raw audio recordings that conflict with signed notes must also be considered due to financial loss risk. KPIs include clinical work Relative Value Units (wRVUs), reduced claim denial rates, and avoided physician turnover costs^66^.

Finally, the Education & Research domain is critical for optimizing workflow support through innovation and defining the overall impact of ambient dictation on efficacy, care processes, and outcomes. This domain supports the ethical foundations of care, enhances evidence-based practice, and drives innovation within clinical workflows. Measurement quantifies core skill acquisition through learner metrics; it may also focus on evaluating AI tool accuracy and error frequency across specialties in simulated versus real-world settings or on standardizing research metrics, such as consistently using assessments like the NASA-TLX or Mini Z^61–64,68^.

This hypothetical TMV scorecard for ambient dictation shows the progression from mission-driven value to strategic priorities and quantified KPIs. This approach integrates ethical considerations throughout. Since TMV also reveals trade-offs and potential conflicts among stakeholder views, it can surface tensions and misaligned priorities. By mapping strategic processes and defining output metrics from a mission-aligned perspective, TMV supports mission-congruent alignment across healthcare organization units.

## Future work

TMV offers a conceptual foundation for addressing AI governance challenges and integrating AI in healthcare, yet further work is required to create a practical, validated framework. Each TMV domain must become actionable strategic priorities for specific organizational contexts, ensuring that unit-level and specialty-specific considerations are included^35,42^. Future work must specify the units of analysis and decision context for TMV, defining measurable KPIs for each domain. Methodological challenges include eliciting and weighting stakeholder priorities, selecting appropriate time horizons for outcome metrics^18,69^, and modeling uncertainty with techniques like MCDA. Implementation research should explore TMV integration within governance, procurement, and AI lifecycle monitoring^9^, considering regulatory constraints such as FDA frameworks^32^. Future work must also investigate trade-offs where mission-value alignment produces tensions, such as AI implementations that improve patient experience but increase clinician workload, equity-oriented deployments with limited financial returns in fee-for-service environments, or mission-driven investments that crowd out other high-impact interventions^11,25^. Finally, the TMV framework requires empirical validation through comparative analyses of AI decisions with and without mission-value integration, longitudinal assessment of TMV-informed outcomes, and cross-institutional studies testing generalizability across healthcare organizations.

AI technologies can benefit patients, healthcare workers, and healthcare organizations, but AI governance must integrate mission-driven values with economic metrics. Current health economic analyses largely focus on cost, and thus risk decoupling strategic decisions from healthcare’s core mission. The five domains of Total Mission Value, Patient Care, Staff Experience, Operations, Economic, and Education and Research, provide an ethically grounded conceptual framework for integrating mission values with business insights and healthcare priorities. Refining these domains into quantifiable metrics and empirically validating TMV are essential for translating this framework into a decision-making tool. Mission-congruent governance of AI technologies through TMV can accelerate AI adoption, enhancing clinical care, organizational capabilities, and economic sustainability.

## Data Availability

No datasets were generated or analysed during the current study.
